# Trajectories of perinatal depression among women living with HIV in Uganda

**DOI:** 10.7189/jogh.14.04147

**Published:** 2024-09-20

**Authors:** Ryan K McBain, Megan S Schuler, Temusa Rukundo, Rhoda K Wanyenze, Glenn J Wagner

**Affiliations:** 1 RAND Corporation, Washington, District of Columbia, USA; 2 RAND Corporation, Santa Monica, California, USA; 3 Makerere University School of Public Health, Kampala, Uganda

## Abstract

**Background:**

Perinatal depression affects one-third of pregnant women living with HIV (WLH). We examined patterns of treatment response to a novel stepped model of depression care among WLH with perinatal depression in Uganda.

**Methods:**

As part of the Maternal Depression Treatment in HIV (M-DEPTH) cluster randomised controlled trial, 191 women were enrolled across four antenatal care clinics assigned to provide stepped care including behavioural and antidepressant therapy (ADT), and another 200 across four clinics assigned to provide usual care. They were assessed for depression severity using the Patient Health Questionnaire (PHQ-9) at enrolment and multiple times over 12 months of follow-up. We used repeated measures latent class analysis (LCA) to identify discrete trajectories of depression symptoms, while multinomial regression analyses measured correlates of class membership.

**Results:**

The LCA identified three trajectories among those in the treatment group: mildly depressed individuals who improved (MiD-I) (n = 143, 75%), moderately depressed individuals who improved (MoD-I) (n = 33, 17%), and moderately depressed individuals who remained depressed (MoD-R) (n = 15, 8%). Membership in MiD-I was associated with lower levels of intimate partner violence at baseline (*P* = 0.04) and month 6 (*P* < 0.001), and less recent trauma exposure (*P* = 0.03) at baseline. At month 6, social support was lowest in MoD-R, while the degree of negative problem-solving orientation was highest (both *P* < 0.001) in this class. The LCA also identified three trajectories among those in the usual care comparison group: mildly depressed (MiD) (n = 62, 31%), moderately depressed (MoD) (n = 71, 35%), and seriously depressed (SiD) (n = 67, 34%), with each experiencing slight improvement. Recent traumas at baseline were highest in SiD (*P* < 0.001); this group also reported the lowest positive problem-solving orientation and highest negative problem-solving orientation (*P* < 0.001) at baseline.

**Conclusions:**

Depression symptom trajectories among women with perinatal depression are related to modifiable factors such as problem-solving orientation and interpersonal dynamics, with the latter including intimate partner violence and social support. Most treatment recipients were characterised by trajectories indicating recovery from depression.

**Registration:**

ClinicalTrials.Gov (NCT03892915).

Mental health conditions are the leading cause of years lived with disability (YLD) in sub-Saharan Africa, with depression accounting for more than 6% of all YLD [1]. In some countries, such as Uganda, this figure is closer to 10% [2]. Rates of depression were previously found to be higher in sub-populations, including individuals living with HIV [3,4] and among pregnant women [5]. For example, an estimated one-third of pregnant women living with HIV (WLH) have a depressive disorder [6], which has been associated with reduced adherence to prevention of mother-to-child transmission (PMTCT) services [7,8] and adverse effects on developmental outcomes for children [9,10].

To address this problem, screening and treatment for depression among WLH at antenatal or HIV care clinics have been proposed in a variety of low-resource settings [11–13]. By leveraging available infrastructure and training existing non-specialist cadres, task-shifted depression care has the potential to reach vulnerable populations at a low cost [14–16]. Additionally, because depression and HIV are chronic conditions, interventions can be monitored longitudinally from the same point of care within the health system.

However, one challenge in the delivery of depression interventions is the observed heterogeneity of treatment effects. Even among interventions that demonstrate significant clinical results, roughly one in three individuals show no meaningful improvement [17,18]. Difficulty predicting the sources of this variation represents a critical barrier to improving clinical outcomes [19,20]. If clinicians could reliably predict which types of individuals would respond less well to treatment, they could augment treatment with specific ancillary interventions that match the needs or characteristics of individual clients.

Over the past decade, researchers have been using person-centered statistical methods with increasing frequency to provide analytical insights regarding heterogeneity within a population [21–23]. In this context, approaches like latent class analysis (LCA) have been designed to identify distinct subgroups (i.e. ‘latent classes’) of individuals who are similar in their outcomes of interest [24,25]. This method could, for example, identify three distinct classes of individuals nested in a sample: ‘responders’ who greatly benefit from behavioural therapy, ‘non-responders’ who show no benefit, and ‘rebounders’ who show a short-term response that later wanes. By determining which types of individuals are most likely to align with specific classes, interventions can be selected and tailored more strategically.

By using LCA, we examined treatment response trajectories corresponding to a stepped care treatment model for perinatal depression among WLH in Uganda. The intervention, known as Maternal DEPression Treatment in HIV (M-DEPTH), was provided to 191 women with varying levels of depression between 2019–22, and their trajectories of depression symptoms were compared to an untreated cohort (n = 200) in the context of a cluster randomised controlled trial [17]. We hypothesised that we would observe discrete trajectories in treatment and control groups, including trajectories defined by significant improvement and non-improvement in depression scores. We also expected that membership in these trajectory groups would correlate with characteristics such as age, partner status, history of intimate partner violence (IPV) and psychosocial characteristics [10,11,18–20].

## METHODS

### Study design

Here we report on a secondary analysis of data collected from the M-DEPTH cluster randomised controlled trial of evidence-based depression care for pregnant WLH in Uganda, previously executed by this research team [17]. The trial was designed to evaluate a stepped model of depression care across eight antenatal care (ANC) clinics operated by the Uganda Ministry of Health – four assigned to the treatment group (treatment protocol described below) and four assigned to the comparison group (i.e. care as usual).

Participants were enrolled during pregnancy and followed up through pregnancy completion and until 18 months postpartum (or six months postpartum in the case of miscarriage, stillbirth, or abortion). Assessments were conducted at enrollment (baseline), as well as at 2-, 6-, 12-, and 18-month postpartum; however, we only included data through six months postpartum in this analysis, as all women were expected to complete this assessment. The institutional review boards of Makerere University in Uganda and the RAND Corporation in Santa Monica, California, provided ethical approval for this study.

### Study participants

To be eligible, participants had to be aged ≥18 years; at week 32 or less of gestation; have an HIV-positive serostatus and be on antiretroviral therapy (ART) for at least four weeks; and have a positive screen for potential depression according to the Patient Health Question (PHQ-9) (i.e. a score of five or higher). Women were deemed ineligible if they were already receiving mental health treatment, or if they were deemed to be at high risk of suicide as indicated by the ninth item of the PHQ-9 – in which case they were referred for immediate specialty care. All participants were recruited when attending routine care visits at ANC clinics between July 2019 and January 2021.

### Treatment protocol

The intervention used a stepped approach, offering psychological and pharmacological interventions for pregnant WLH who screened positive for depression. Specifically, women were screened by peer mothers (lay health workers at ANC clinics) using the two-item version of the Patient Health Questionnaire (PHQ-2). Those who screened positive (PHQ-2 > 0) were referred to a nurse who administered the full nine-item PHQ-9.

Those with moderate depression (PHQ-9 score: 10–19) were recommended problem-solving therapy (PST), while those with severe depression (PHQ-9 score: >20) were recommended antidepressant therapy (ADT), consistent with the World Health Organization’s mhGAP Intervention Guide recommendations [21]. Ultimately, patients had the autonomy to choose their preferred treatment modality after consultation with their provider. Women whose depressive symptoms were mild (PHQ-9 score: 5–9) and not warranting treatment received depression psychoeducation and monthly monitoring of depression at routine ANC visits; treatment would be offered at a later time point if depression increased.

Peer mothers received training to implement manualised individual PST over three biweekly core sessions, followed by up to four ancillary monthly sessions (i.e. those continuing to experience depressive symptoms could receive a maximum of seven sessions). ADT was administered and monitored by nurses, no earlier than the second trimester to balance benefits with risks to the developing foetus. Fluoxetine (a selective serotonin reuptake inhibitor) was the first-line medication at a starting daily dose of 20 mg, while imipramine (a tricyclic antidepressant) was the second-line option at a starting daily dose of 50 mg (increased to 75 mg after the first week). At monthly follow-up visits, measures of treatment response and assessment of side effects guided changes to dosage or medication. Further details on treatment protocols can be found elsewhere [17]. Mental health specialists hired by the study provided monthly supervision to the peer mothers and nurses.

The comparison condition (usual care) is characterised in Ugandan ANC clinics as a referral to a mental health specialist at a district or regional referral hospital for patients exhibiting significant symptoms. There are no mental health-specific services available throughout Uganda at ANC clinics. For WLH, these clinics offer family support groups for psychosocial support and education to support pregnancy and postpartum care, as well as PMTCT adherence. These typically comprise 24-monthly group sessions (each lasting two hours) that occur from early pregnancy through 18 months postpartum.

### Measures

The following measures were assessed with a computer-assisted, interviewer-administered survey, or chart abstraction as noted. All measures were collected at baseline and two- and six-months postpartum, unless otherwise noted.

#### PHQ-9

The primary outcome measure was the PHQ-9 [22], which had been adapted and validated in the Ugandan context, as well as translated to Luganda, the local language where the trial was conducted [23,24]. The instrument contains nine items that ask individuals to self-report the frequency of nine depression symptoms over the past two weeks on a four-point ordinal scale: not at all (0), several days (1), more than half the days (2), nearly every day (3). A score of ten or above indicates probable major depressive disorder, consistent with prior research evaluating the PHQ in the Ugandan context [25]. The PHQ-9 was administered to all participants at each study assessment for both the intervention and usual care groups (i.e. two and six months postpartum, or approximately six and 12 months after baseline). Women in the intervention group also received assessments at months three and nine after baseline as part of treatment monitoring.

#### Sociodemographic characteristics

The surveys collected the following background information on the participants: characteristics related to demographics (age, any secondary education, relationship status) and pregnancy-related characteristics (length of gestation at baseline, pregnancy outcome, whether pregnancy was planned).

#### HIV characteristics

Research team members abstracted additional information from patients’ health records, such as confirmation and date of HIV diagnosis, and duration of time on ART. They assessed HIV viral load based on blood assays they had collected at baseline. Knowledge of aspects of PMTCT (e.g. use of ART and breastfeeding approaches to limit transmission risks) was measured as the sum of correct yes/no responses across seven items, while negative attitudes towards the use of PMTCT recommended practices were assessed as the mean five-point rating of agreement across four items – all developed by the study team.

#### Partner/relationship measures

For those with a primary partner at the time of the assessment, we assessed the presence of IPV and partner support for pregnancy. Specifically, we determined IPV victimisation experienced in the past six months through seven items from the Development and Household Survey [26] that assessed emotional, physical, controlling, and sexual forms of IPV. Afterwards, a cumulative count (ranging from 0–7) of the number of ‘yes’ responses was calculated to determine the sum of IPV types experienced. Partner support during pregnancy was assessed with six items from the Social Support Effectiveness scale, which measured aspects of task-related, informational, and emotional support during pregnancy [27].

#### Psychosocial measures

General social support was assessed using the modified 10-item Duke-University of North Carolina Functional Social Support Questionnaire [28]. Experience of traumatic events during childhood (before 17 years of age) and recently (past three years) was assessed using modified versions of the Childhood Trauma Questionnaire and Recent Trauma Events Scale, both developed by Pennebaker et al. [29]. Problem-solving orientation (positive problem-solving, negative problem-solving) and avoidant problem-solving style were assessed with the five-item subscale measures of each construct from the Social Problem Solving Inventory-Revised [30]. Internalized HIV stigma was assessed with an eight-item scale (e.g. ‘I am ashamed that I am HIV positive’) developed by Kalichman et al. [31]. Health functioning was assessed with 16 items from the Medical Outcomes Study – HIV survey [32].

### Statistical analysis

We conducted repeated measures LCA to identify groups of individuals with similar longitudinal patterns of depression symptoms as measured by the PHQ-9. As a type of finite mixture modelling, LCA assumes an underlying data structure of mutually exclusive categorical subgroups (i.e. classes) [33]. We implemented repeated measures LCA models separately for women in the treatment group (n = 191) and those in the control group (n = 200).

For the treatment group, LCA indicators comprised measures of PHQ-9 at baseline and aggregated indicators of the mean PHQ-9 values measured at three, six, nine, and 12 months after enrolment. For the comparison group, LCA indicators comprised PHQ-9 values at baseline and six and 12 months after enrolment. There was individual variance regarding the exact timing of the measurement, so each of these time periods has a +/− one-month window for the timing of the administration. For both the treatment and comparison groups, we compared LCA models with two to five classes based on multiple fit statistics, including Akaike information criterion (AIC), Bayesian information criterion (BIC), sample-size adjusted BIC, and entropy, as well as assessing minimum class size. We report class-specific mean values of the PHQ-9 at each time point to characterise the trajectories identified for both the treatment and comparison groups.

Finally, to investigate systematic differences in women across each of the trajectories, we examined associations between trajectory class membership with baseline and time-varying sociodemographic and HIV characteristics, as described above. We used separate regression models for each covariate to test for omnibus differences means across classes and subsequently performed multivariable multinomial regressions to examine the magnitude of individual relationships when adjusted for covariates: all sociodemographic, HIV and partner/relationship measures. We conducted all latent class modelling in Mplus, version 8.7 (Linda and Bengt Muthén, Los Angeles, CA, USA) and all other analyses in Stata, version 18.0 (StataCorp LLC, College Station, TX, USA).

## RESULTS

In total, 191 individuals were enrolled at sites randomised to provide the treatment condition (M-DEPTH) and 200 at sites randomised to the control condition. Groups were balanced on most characteristics, including age, education, stage of pregnancy, and HIV characteristics (**Table 1**).

**Table 1:** Sample characteristics

Characteristics	Treatment group (n = 191), %	Comparison group (n = 200), %
Age category in years		
*Up to 20*	11.0	13.5
*21–25*	27.7	27.5
*26–30*	33.5	28.5
*31–35*	19.4	18.5
*≥36*	8.4	12.0
Demographic characteristics		
*Any secondary education*	41.4	30.5
*In a relationship*	80.6	84.0
*Any childhood trauma*	81.7	86.0
Pregnancy characteristics		
*Planned pregnancy*	45.8	43.2
*Successfully delivered*	93.2	90.3
*Clinical characteristics*		
*Newly diagnosed with HIV*	21.5	20.0
*Good ART adherence*	74.9	76.3
*Depressed at treatment initiation*	63.9	72.5
Trimester		
*1st*	17.8	11.5
*2nd*	57.6	70.5
*3rd*	24.6	18.0

ART – antiretroviral therapy, HIV – human immunodeficiency virus

### Model selection

We selected the three-class model to characterise the treatment group. We observed a modest reduction in the AIC from the two-class solution to the three-class solution (4460 vs 4443), as well as in the BIC (4461 vs 4445). We also selected the three-class model to characterise the comparison group. Here, the BIC was lower in the three-class model (3477 vs 3481), while the AIC was higher (3431 vs 3422). Entropy did not differ meaningfully (0.704 vs 0.709). Therefore, we selected the three-class model for parsimony.

### Latent class characteristics

#### Treatment group

**Figure 1:**
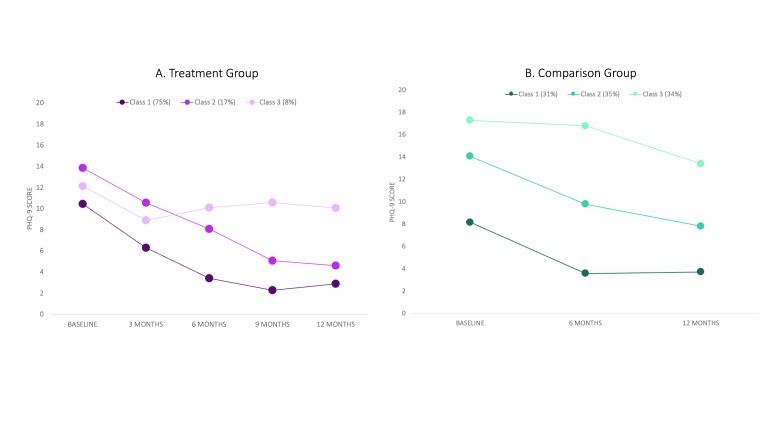
Latent class trajectories in treatment and comparison groups. Each point represents the mean value of the class at the particular time point. Treatment group represents those assigned to M-DEPTH; comparison group represents those assigned to care as usual.

**Figure 1**, Panel A shows the PHQ-9 trajectory for each latent class (labelled as MiD-I, MoD-I, and MoD-R for descriptive purposes) in the treatment group.

##### MiD-I (class 1)

MiD-I comprised 75% of those in the intervention group (n = 143). As this label indicates, these individuals’ trajectories had a steady linear decline in depression symptoms from baseline to month 6, after which depression symptoms remained low through month 12. Among those in this group, 30% received psychoeducation, 45% received PST, and 25% received ADT.

##### MoD-I (class 2)

MoD-I represented 17% of individuals in the intervention group (n = 33). On average, these individuals started with higher depression symptom scores at baseline than the MiD-I – with an average score of 14 on the PHQ-9 compared to 10 – and showed a gradual decline through month 9, after which depressive symptoms remained slightly above those in the MiD-I class. Among those in this group, 12% received psychoeducation, 33% received PST, and 55% received ADT.

##### MoD-R (class 3)

MoD-R had 8% of intervention group members (n = 15). Although this was the smallest group, these individuals experienced the least diminution in PHQ-9 scores, with only slight improvements from baseline to three months, after which average depression symptom levels increased slightly and remained elevated. Among those in this group, 27% received psychoeducation, 27% received PST, and 47% received ADT. While this group had an average moderate level of depression at baseline, inclusion was more based on the overall trajectory of scores over time. Therefore, even those with relatively low PHQ-9 scores at baseline (i.e. those receiving only psychoeducation) could have been placed in this group if their depression levels did not improve or even worsened over time.

#### Comparison group

**Figure 1**, Panel B shows the PHQ-9 trajectory for each latent class (labelled MiD, MoD, and SiD for descriptive purposes) in the non-treatment control group. In each of these classes, there were varying levels of improvement experienced by the class as a whole over time.

##### MiD (class 1)

MiD comprised 31% of those in the control group (n = 62). These individuals reported relatively low depression symptom severity at baseline (average PHQ-9 score was 8), with modest improvement in symptoms at six and 12 months after baseline.

##### MoD (class 2)

MoD represented 35% of those in the control group (n = 71). This trajectory was characterized by moderate depression scores at baseline (average PHQ-9 of 14) with gradual symptom improvement at six and 12 months after baseline.

##### SiD (class 3)

SiD covered 34% of individuals in the control group (n = 67) with little change through month 6, followed by modest improvement 12 months after baseline. These individuals reported high depression symptom severity at baseline (average PHQ-9 score was 17).

### Latent class regression models

#### Treatment group

We conducted latent class regression to examine differences across classes with respect to baseline and time-varying characteristics (**Table 2**). We found significant differences in the sum of types of IPV victimisation experienced, as well as controlling and physical IPV across the three classes. Specifically, those in the MiD-I reported the lowest mean scores for the sum of IPV types at baseline (*P* = 0.04) and month 6 (*P* < 0.001), for controlling IPV at month 6 (*P* < 0.001), and for physical IPV at baseline (*P* = 0.04). Likewise, the baseline number of recent traumas (*P* = 0.01) and any recent traumas (*P* = 0.03) were lower in the MiD-I group.

**Table 2:** Mean values for independent measures by latent class in treatment group

	MiD-I	MoD-I	MoD-R	*P-*value*
**Experienced IPV**				
Sum of IPV types (range: 0–7)				
*Baseline*	3.3	4.1	4.3	0.04
*Month 6*	3.0	4.2	4.3	<0.001
*Month 12*	3.1	3.8	3.8	0.17
Controlling IPV (range: 0–4)				
*Baseline*	2.3	2.8	2.8	0.08
*Month 6*	2.2	3.0	2.9	<0.001
*Month 12*	2.3	2.7	2.7	0.34
Physical IPV (range:0-2)				
*Baseline*	0.4	0.5	0.9	0.04
*Month 6*	0.3	0.4	0.7	0.12
*Month 12*	0.3	0.6	0.3	0.15
**Problem-solving orientation (range: 0–20)**				
Negative orientation				
*Baseline*	10.0	11.6	11.2	0.13
*Month 6*	9.4	11.0	13.5	<0.001
*Month 12*	8.7	9.7	11.7	0.04
Positive orientation				
*Baseline*	14.1	14.3	13.3	0.71
*Month 6*	15.7	14.7	14.8	0.27
*Month 12*	15.5	15.6	13.9	0.36
Avoidant orientation				
*Baseline*	5.8	6.7	6.2	0.64
*Month 6*	5.3	6.8	7.5	0.14
*Month 12*	4.9	6.2	7.1	0.15
**Partner support**				
Pregnancy support (range: 0–5)				
*Baseline*	2.7	2.6	2.1	0.14
*Month 6*	3.1	3.0	2.6	0.37
*Month 12*	3.1	3.1	2.8	0.67
ANC support (range: 0–9)				
*Baseline*	3.5	3.3	2.5	0.36
*Month 6*	3.6	3.9	2.1	0.12
*Month 12*	3.6	3.5	2.1	0.20
**General social support (range: 0–4)**				
Baseline	2.4	2.1	2.3	0.18
Month 6	2.7	2.4	2.3	0.03
Month 12	2.6	2.4	2.0	<0.001
**Trauma**				
Any recent trauma – baseline	79.7%	97.0%	93.3%	0.03
Number of recent traumas – baseline	1.9	2.7	2.8	0.01
Any childhood trauma – baseline	78.3%	90.9%	93.3%	0.12
**PMTCT (range: 0–7)**				
PMTCT knowledge – baseline	4.1	3.8	3.9	0.49
PMTCT attitudes – baseline	4.2	4.2	3.9	0.11
**Health (range: 0–100)**				
Health-related quality of life – baseline	57.5	46.4	47.7	<0.001
**Stigma (range: 0–5)**				
HIV-related stigma – baseline	3.4	3.7	3.4	0.22

ANC – antenatal care, HIV – human immunodeficiency virus, IPV – intimate partner violence, MiD-I – mildly depressed improvers, MoD-I – moderately depressed improvers, MoD-R – moderately depressed remain depressed, PMTCT – prevention on mother-to-child transmission of HIV

*Omnibus F-test.

We also observed that general social support was lowest in the MoD-R group and highest in the MiD-I group – both at month 6 (*P* = 0.03) and month 12 (*P* < 0.001). Similarly, negative problem-solving orientation was highest in the MoD-R group and lowest in the MiD-I group at both month 6 (*P* < 0.001) and month 12 (*P* = 0.04).

We further found that average health-related quality of life was highest at baseline among those in the MiD-I group (mean = 57.5), compared to those in the MoD-I group (mean = 46.4) and in the MoD-R group (mean = 47.7; *P* < 0.001).

#### Comparison group

Latent class regressions also identified a wide range of differences among those in the comparison group latent classes (**Table 3**). With respect to IPV, those in the MoD group reported the lowest scores pertaining to the sum of IPV types experienced at months six and 12 (*P* < 0.001), controlling IPV at month 6 (*P* = 0.02) and month 12 (*P* < 0.001), and physical IPV at month 6 (*P* < 0.001) and month 12 (*P* < 0.001). Number of recent traumas at baseline was lowest among those in MoD and highest among those in SiD (*P* < 0.001).

**Table 3:** Mean values for independent measures by latent class in the control group

	MiD-I	MoD-I	MoD-R	*P-*value*
**Experienced IPV**				
Sum of IPV types (range: 0–7)				
*Baseline*	3.6	3.1	4.1	0.07
*Month 6*	3.9	2.7	4.3	<0.001
*Month 12*	3.6	2.4	4.5	<0.001
Controlling IPV (range: 0–4)				
*Baseline*	2.3	2.2	2.5	0.39
*Month 6*	2.7	2.0	2.7	0.02
*Month 12*	2.4	1.9	2.9	<0.001
Physical IPV (range: 0-2)				
*Baseline*	0.5	0.5	0.7	0.11
*Month 6*	0.4	0.3	0.8	<0.001
*Month 12*	0.6	0.2	0.7	<0.001
**Problem solving orientation (range: 0–20)**				
Negative orientation				
*Baseline*	12.3	10.1	15.1	<0.001
*Month 6*	11.2	10.2	13.6	<0.001
*Month 12*	10.9	9.0	13.5	<0.001
Positive orientation				
*Baseline*	13.9	14.3	12.1	0.01
*Month 6*	14.8	15.7	13.6	0.02
*Month 12*	15.0	15.6	13.6	0.02
Avoidant orientation				
*Baseline*	8.3	7.0	9.4	0.03
*Month 6*	6.7	6.7	7.3	0.80
*Month 12*	6.1	6.6	6.5	0.81
**Partner support**				
Pregnancy support (range: 0–5)				
*Baseline*	2.5	2.8	1.9	<0.001
*Month 6*	2.6	3.2	2.3	<0.001
*Month 12*	2.8	3.3	2.3	<0.001
ANC support (range: 0–9)				
*Baseline*	3.6	3.9	2.0	<0.001
*Month 6*	3.7	4.4	2.3	<0.001
*Month 12*	3.6	3.2	2.4	0.08
**General social support (range: 0–4)**				
Social support				
*Baseline*	2.2	2.6	1.8	<0.001
*Month 6*	2.2	2.9	1.8	<0.001
*Month 12*	2.3	2.8	1.8	<0.001
**Trauma**				
Any recent trauma – baseline	0.8	0.8	0.9	0.90
Number of recent traumas – month 6	3.0	1.9	4.3	<0.001
Any childhood trauma – month 12	0.9	0.8	0.9	0.49
**PMTCT (range: 0–7)**				
PMTCT knowledge – baseline	3.9	4.4	3.5	<0.001
PMTCT attitudes – baseline	4.3	4.1	4.1	<0.001
**Health (range: 0–100)**				
Health-related quality of life – baseline	46.5	60.2	38.4	<0.001
**Stigma (range: 0–5)**				
HIV-related stigma – baseline	3.6	3.2	3.7	0.01

ANC – antenatal care, HIV – human immunodeficiency virus, IPV – intimate partner violence, MiD-I – mildly depressed improvers, MoD-I – moderately depressed improvers, MoD-R – moderately depressed remain depressed, PMTCT – prevention on mother-to-child transmission of HIV

*Omnibus F-test.

Positive problem-solving orientation was lowest among those in SiD at baseline (*P* = 0.01), month 6 (*P* = 0.02), and month 12 (*P* = 0.02). Conversely, negative problem-solving orientation was highest among those in SiD at all three time points (*P* < 0.001).

Scores on PMTCT knowledge were lowest at baseline among those in the SiD class (*P* < 0.001), with higher scores on negative PMTCT attitudes in the MiD class at baseline (*P* < 0.001). The lowest levels of perceived partner support in pregnancy were reported by those in the SiD class at all three time points (*P* < 0.001). We observed the same pattern at baseline (*P* < 0.001) and month 6 (*P* < 0.001) for perceived partner support of ANC.

### Multivariable multinomial regression models

Among those in the treatment group, we observed that the MoD-I (*P* < 0.01) and MoD-R (*P* < 0.01) classes reported lower baseline health-related quality of life, compared to the MiD-I class. Likewise, the MoD-R class reported more negative attitudes towards PMTCT (*P* = 0.02) and higher levels of physical IPV (*P* = 0.04) compared to those in the MiD-I class at baseline (Tables S1–2 in the **Online Supplementary Document**).

Among those in the control group, the MoD class at baseline reported lower PMTCT knowledge (*P* = 0.003) and health-related quality of life (*P* < 0.001) compared to those in the MiD class. Similarly, those in the SiD class reported lower PMTCT knowledge (*P* = 0.001) and health-related quality of life (*P* < 0.001) compared to the MiD class at baseline. The SiD class at baseline also reported a higher negative problem-solving orientation compared to the MiD class at baseline (*P* = 0.01). The MoD class also reported fewer negative attitudes towards PMTCT compared to the MiD class at baseline (*P* = 0.008).

## DISCUSSION

In this study, we found that three trajectories characterised the depression symptom profiles of Ugandan WLH with perinatal depression assigned to treatment: a large majority had mild depression at baseline that improved over the course of treatment (class 1: MiD-I); a smaller group had moderate depression at baseline that also improved over the course of treatment (class 2: MoD-I); and a yet smaller group had moderate depression that was relatively unaffected by treatment (class 3: MoD-R). We also observed three trajectories for women in the comparison group, which were evenly split at one-third each: those with mild depression (class 1: MiD), those with moderate depression (class 2: MoD), and those with serious depression (class 3: SiD), each of which improved slightly over time.

With respect to the treatment group, we found that a large plurality showed substantive improvements toward recovery: 92% were classified as improvers (75% were ‘mild depression, improvers’ and 17% were ‘moderate depression, improvers’). Other LCAs examining treatment responses to mental health interventions have reported that a sizable majority of participants are classified by trajectories of improvement [34–36]. Consistent with prior literature, we also find that participants with less exposure to IPV and traumatic events reported lower levels of depression and greater symptom improvement [13,18,37]. For example, a meta-analysis primarily involving studies based in sub-Saharan Africa found that pregnant WLH who had experienced IPV had nearly double the odds of experiencing perinatal depression, compared to their counterparts who did not experience IPV [18]. In Uganda, rates of IPV are estimated to be 45% for lifetime prevalence among women [38] and have been reported to be as high as 56% among married women [39]. Interventions targeting reductions in IPV have demonstrated modest success [40,41], offering a potential opportunity to integrate IPV reduction strategies into depression care for pregnant women.

We also observed that social support was lower among those in the MoD-R group that showed little symptom recovery, along with higher levels of a negative orientation to problem-solving. Similar to IPV, these may represent opportunities for augmenting the M-DEPTH intervention, at least among those identified as treatment-resistant. The behavioural intervention component of M-DEPTH (PST) contains a module on fostering social support [42]; however, the practicalities of this may be constrained in rural communities where individuals feel isolated or stigmatized for HIV and mental health needs [43,44]. Likewise, other forms of PST employ a multi-step process for problem-solving, but do not include explicit discussion of problem-solving orientations that are adaptive or maladaptive.

We distinguished trajectories among those in the usual care comparison group by baseline depression severity levels (mild, moderate, serious); participants within these classifications also experienced some improvement in depressive symptoms over time, albeit to a lesser extent than those in the treatment group. This improvement is likely to reflect regression to the mean [45] as well as potential benefits of usual care. For those with mild symptoms at baseline, this implied a reduction that led participants to conclude the trial at a sub-clinical depression threshold; this was not the case for the other two classes. Similar to those in the treatment group, we observed that IPV distinguished depression severity over the course of the trial among those in the comparison group, as did recent exposure to trauma and problem-solving orientation.

Among those in the comparison group, knowledge and negative attitudes regarding PMTCT distinguished two trajectories: those with SiD had the lowest PMTCT knowledge scores, and those with MiD had the highest scores on negative PMTCT attitudes. This means that those with serious depression showed less knowledge about PMTCT, while those with mild depression had more negative attitudes. The clinical trial was not powered to detect differences in vertical transmission of HIV among those with different baseline levels of depression symptoms; however, this would be an important line of inquiry for future, larger trials.

Lastly, we observed that partner engagement shaped depression trajectories among those in the comparison group, whereby self-reported partner support for ANC and pregnancy overall was lowest among those in the SiD classification. Prior research has shown that discordant views among partners on pregnancy and health care are associated with household disputes, worse mental health, and worse outcomes for the child following pregnancy [19,27,46,47]. While forms of depression care that seek to engage partners directly may be one way of addressing this [48], family-based models of mental health care in low-resource settings have documented practical challenges with implementation, such as time constraints of family members [49,50].

We note several study limitations. First, measurement intervals and frequencies were not equivalent between the treatment and comparison groups, limiting our ability to make direct comparisons, and we instead focused on each group separately. Second, latent classes with small numbers of individuals had limited statistical power to make direct comparisons in multinomial regression models; it was primarily for this reason that we relied on omnibus tests when comparing differences across latent classes overall. Third, we lacked detailed data regarding HIV treatment (e.g. specific ART regimens), so we could not examine potential treatment differences across classes. Lastly, certain social determinants of health – such as household income – were uniformly low in the research setting. Limited heterogeneity on such factors precluded our ability to test the relationships between such variables and depression symptom severity.

## CONCLUSIONS

Despite these limitations, our study helps identify potentially modifiable factors that are associated with depression symptom trajectories, and it catalogues community dynamics that could be influenced through an updated version of M-DEPTH or other care models for perinatal depression. We also found that a large plurality of women assigned to M-DEPTH (>90%) had trajectories in which depression symptoms were below clinically diagnostic thresholds by 12-month follow-up. Future research might build off of these findings by refining intervention design or by examining longer-term impacts of such interventions on parents, as well as infant development.

## Additional material


Online Supplementary Document
