# Supplementary material for: Trajectories of perinatal depression among women living with HIV in Uganda
Source: J Glob Health. 2024 Sep 20;14:04147. doi: 10.7189/jogh.14.04147 (PMC11413615; doi:10.7189/jogh.14.04147)
Supplement: Online Supplementary Document [file jogh-14-04147-s001.pdf]

ONLINE SUPPLEMENTARY MATERIALS

Table S1. Multivariable Multinomial Regression Results, Treatment Group

Variable Name	Latent Class 2 vs. Latent Class 1		Latent Class 3 vs. Latent Class 1	
	**Coefficient (SE)**	**95 CI**	**Coefficient (SE)**	**95 CI**
Experienced Intimate Partner Violence (IPV)				
Sum of IPV types	0.08 (0.44)	-0.77, 0.94	-1.18 (0.80)	-2.75, 0.38
Controlling IPV	0.27 (0.60)	-0.92, 1.45	2.13 (1.18)	-0.17, 4.43
Physical IPV	-0.32 (0.59)	-1.48, 0.85	2.29* (1.09)	0.16, 4.41
Problem Solving Orientation				
Negative Orientation	0.04 (0.07)	-0.10, 0.18	0.03 (0.14)	-0.24, 0.30
Positive Orientation	0.12 (0.07)	-0.03, 0.26	-0.16 (0.12)	-0.41, 0.08
Avoidant Orientation	0.08 (0.06)	-0.04, 0.19	-0.08 (0.13)	-0.34, 0.17
Partner Support				
Pregnancy Support	0.03 (0.36)	-0.66, 0.73	-1.37 (0.76)	-2.87, 0.12
Antenatal Care Support	0.02 (0.14)	-0.26, 0.30	-0.19 (0.25)	-0.68, 0.29
General Support				
Social Support	0.22 (0.56)	-0.88, 1.33	2.56 (1.29)	0.03, 5.09
Trauma				
Any Recent Trauma	1.98 (1.39)	-0.75, 4.71	1.57 (2.57)	-3.47, 6.60
Number of Recent Traumas	-0.03 (0.17)	-0.36, 0.31	-0.31 (0.29)	-0.87, 0.25
Any Childhood Trauma	2.20 (1.31)	-0.37, 4.78	3.95 (2.33)	-0.61, 8.51
PMTCT				
PMTCT Knowledge	0.07 (0.24)	-0.39, 0.54	-0.02 (0.4)	-0.80, 0.76
PMTCT Attitudes	0.88 (0.78)	-0.65, 2.41	-2.21* (0.90)	-3.97, -0.44
Health				
Health-Related Quality of Life	-0.12** (0.04)	-0.19, 0.05	-0.22** (0.08)	-0.38, -0.07
Stigma				
HIV-Related Stigma	-0.03 (0.34)	-0.70, 0.63	-0.43 (0.57)	-1.54, 0.69

Notes: Class 1: Mildly depressed—improvers (MiD-I); Class 2: Moderately depressed—improvers (MoD-I); Class 3: Moderately depressed—remains depressed (MoD-R); 95% CI: 95% confidence interval. Note: all measures assessed at baseline. *p<0.05; **p<0.01; ***p<0.001.

Table S2. Multivariable Multinomial Regression Results, Comparison Group

Variable Name	Latent Class 2 vs. Latent Class 1		Latent Class 3 vs. Latent Class 1	
	**Coefficient (SE)**	**95 CI**	**Coefficient (SE)**	**95 CI**
Experienced Intimate Partner Violence (IPV)				
Sum of IPV types	0.49 (0.41)	-0.31, 1.29	0.33 (0.50)	-0.66, 1.31
Controlling IPV	-0.31 (0.58)	-1.44, 0.83	-0.29 (0.70)	-1.66, 1.08
Physical IPV	-1.05 (0.58)	-2.18, 0.08	-0.47 (0.68)	-1.81, 0.87
Problem Solving Orientation				
Negative Orientation	0.11 (0.07)	-0.02, 0.25	0.21** (0.08)	0.04, 0.38
Positive Orientation	0.05 (0.07)	-0.08, 0.18	-0.08 (0.08)	-0.24, 0.08
Avoidant Orientation	0.00 (0.05)	-0.10, 0.10	0.07 (0.07)	-0.07, 0.20
Partner Support				
Pregnancy Support	-0.01 (0.40)	-0.79, 0.78	-0.15 (0.50)	-1.12, 0.83
Antenatal Care Support	0.13 (0.13)	-0.13,0.40	-0.06 (0.17)	-0.39, 0.26
General Support				
Social Support	-0.17 (0.43)	-1.02, 0.68	0.16 (0.54)	-0.90, 1.22
Trauma				
Any Recent Trauma	0.22 (0.95)	-1.64, 2.07	0.53 (1.17)	-1.7, 2.83
Number of Recent Traumas	0.00 (0.15)	-0.29,0.29	0.05 (0.15)	-0.25, 0.36
Any Childhood Trauma	0.19 (0.78)	-1.34, 1.72	0.29 (1.07)	-1.81, 2.39
PMTCT				
PMTCT Knowledge	-0.68** (0.23)	-1.13, -0.28	-0.92** (0.28)	-1.47, -0.37
PMTCT Attitudes	2.15** (0.81)	0.56, 3.74	-0.78 (1.27)	-3.27, 1.70
Health				
Health-Related Quality of Life	-0.14*** (0.03)	-0.20, -0.08	-0.20*** (0.04)	-0.28, -0.13
Stigma				
HIV-Related Stigma	0.13 (0.27)	-0.39, 0.65	0.25 (0.32)	-0.38, 0.88

Notes: Class 1: Mildly Depressed (MiD); Class 2: Moderately Depressed (MoD); Class 3: Seriously Depressed (SiD); 95% CI: 95% confidence interval. Note: all measures assessed at baseline. *p<0.05; **p<0.01; ***p<0.001.
